# Spatial Resolution Enhancement Framework Using Convolutional Attention-Based Token Mixer

**DOI:** 10.3390/s24206754

**Published:** 2024-10-21

**Authors:** Mingyuan Peng, Canhai Li, Guoyuan Li, Xiaoqing Zhou

**Affiliations:** Land Satellite Remote Sensing Application Center, MNR, Beijing 100048, China; licanhai@lasac.cn (C.L.); liguoyuan@lasac.cn (G.L.); zhouxiaoqing@lasac.cn (X.Z.)

**Keywords:** data fusion, spatial resolution enhancement, convolutional attention, token mixer

## Abstract

Spatial resolution enhancement in remote sensing data aims to augment the level of detail and accuracy in images captured by satellite sensors. We proposed a novel spatial resolution enhancement framework using the convolutional attention-based token mixer method. This approach leveraged spatial context and semantic information to improve the spatial resolution of images. This method used the multi-head convolutional attention block and sub-pixel convolution to extract spatial and spectral information and fused them using the same technique. The multi-head convolutional attention block can effectively utilize the local information of spatial and spectral dimensions. The method was tested on two kinds of data types, which were the visual-thermal dataset and the visual-hyperspectral dataset. Our method was also compared with the state-of-the-art methods, including traditional methods and deep learning methods. The experiment results showed that the method was effective and outperformed state-of-the-art methods in overall, spatial, and spectral accuracies.

## 1. Introduction

Spatial resolution enhancement in remote sensing data is a crucial research area that aims to augment the level of detail and accuracy in images captured by sensors in airborne or satellite platforms. With advancements in remote sensing technology, there has been a growing demand for high-resolution image data, as they enable more effective analysis and interpretation for a wide range of applications, including land cover classification, environmental monitoring, and urban planning.

Despite the significant progress made in remote sensing data acquisition, the inherent limitations of sensor capabilities, such as spatial constraints and data transmission bandwidth, often result in images not being able to possess high spatial and high spectral resolutions at the same time. For example, hyperspectral data tend to possess high spectral resolutions, often reaching 10 nm and possessing hundreds of bands, but low spatial resolution often reaches 10 m or more. This imposes challenges on researchers and practitioners, as they seek to obtain comprehensive and informative image data for their analysis.

The spatial resolution enhancement of remote sensing data has been a significant research area, with various methods proposed to address this challenge. One commonly adopted approach is data fusion, which aims to utilize the different resolution characteristics from two images and fuse them into one image so that the composition can include the highest resolutions of both. One way to enhance the spatial resolution is the spatial–spectral fusion, which fuses the high-spatial–low-spectral-resolution image and low-spatial–high-spectral-resolution image to obtain a high-spatial–high-spectral-resolution image. Data fusion has shown promising results in improving the visual quality and fine details in remote sensing images. To address this issue, numerous spatial resolution enhancement methods for hyperspectral data have been proposed in literature, leveraging various techniques such as interpolation, super-resolution, and deep learning approaches.

Wei et al. developed a Bayesian algorithm for fusing hyperspectral and multispectral images, demonstrating efficiency compared to state-of-the-art fusion techniques [1]. Gao et al. proposed a transformer-based baseline, CSMFormer [2], for HS/MS fusion and classification, improving data quality and classification precision. Wang et al. introduced SSCFNet [3], a spatial–spectral cross-fusion network for remote sensing change detection, outperforming other state-of-the-art methods. Chen et al. proposed EFCOMFF-Net [4] for remote sensing image scene classification, enhancing feature correlation for improved representation ability, showcasing advancements in fusion, classification, and change detection for improved data quality and representation. Peng et al proposed a method using convolutional neural networks (CNN) to enhance the spectral resolution of multispectral data, aiming to improve the ability to distinguish different materials within an image by refining its spectral information [5]. Xie et al. introduced a 1D-convolutional neural network for hyperspectral and multispectral data fusion, emphasizing the extraction of spectral features for improved performance [6]. Wu et al. highlighted the success of transformer-based models, showing comparable or superior performance, compared to other neural network types [7]. Bu et al. proposed a hybrid convolution and spectral symmetry preservation network for hyperspectral super-resolution to improve the spatial resolution of hyperspectral data [8]. Luan et al. used a cascade of multiple-scale spatial contextual modules and spatial–spectral fusion transformer modules to reconstruct spectral information from RGB images to obtain high-resolution hyperspectral data [9].These studies collectively illustrate the diverse approaches and techniques used in the spatial resolution enhancement of remote sensing data, encompassing super-resolution reconstruction, weakly supervised learning, and attention mechanisms. By leveraging deep learning and attention-based methods, researchers have made significant progress in enhancing the spatial resolution and extracting valuable information from remote sensing imagery.

Various studies highlight the effectiveness of attention mechanisms in improving tasks, such as image classification, scene classification, object detection, and landside extraction in remote sensing applications. These mechanisms enhance model performance and accuracy in analyzing remote sensing data. Feng et al. introduced a high-precision remote sensing image classification method using machine learning methods for automatic classification and recognition of ground objects [10]. Li et al. proposed a few-shots remote scene classification method based on attention mechanisms for the semantic understanding of remote sensing images [11]. However, most of these methods using transformer suffer from a high-latency dilemma. For example, the multi-head self-attention (MHSA) mechanism often comes with quadratic complexity, with respect to token length [12]. This greatly impedes the real applications of these methods.

In order to enhance the deployment without dragging down the accuracies, we propose a novel spatial resolution enhancement framework for remote sensing data utilizing a convolutional attention-based token mixer (SRE-CATM). By incorporating attention mechanisms and token-mixing operations into the resolution enhancement process, our framework aims to enable selective feature attention and enhanced information fusion at the token level. This approach effectively leverages spatial context and semantic information, facilitating improved visual quality and enhanced accuracy in the resulting images. This can solve the problem of insufficiency of spatial resolution of hyperspectral or infrared thermal images, which can improve the accuracies of real-world applications involving fine texture extraction. Our method could fall into the category of spatial–spectral fusion, yet it can also apply to non-hyperspectral data, so we present a framework of spatial resolution enhancement.

The subsequent sections of this paper are structured as follows: Section 2 presents the methodology, outlining the proposed framework’s architecture and operation in detail. In Section 3, we present extensive experimental results and comparisons with other state-of-the-art methods to validate the effectiveness of our approach. Finally, Section 4 concludes the paper with a discussion of the findings and future research directions for spatial resolution enhancement in remote sensing data.

## 2. Methods

**Figure 1:**
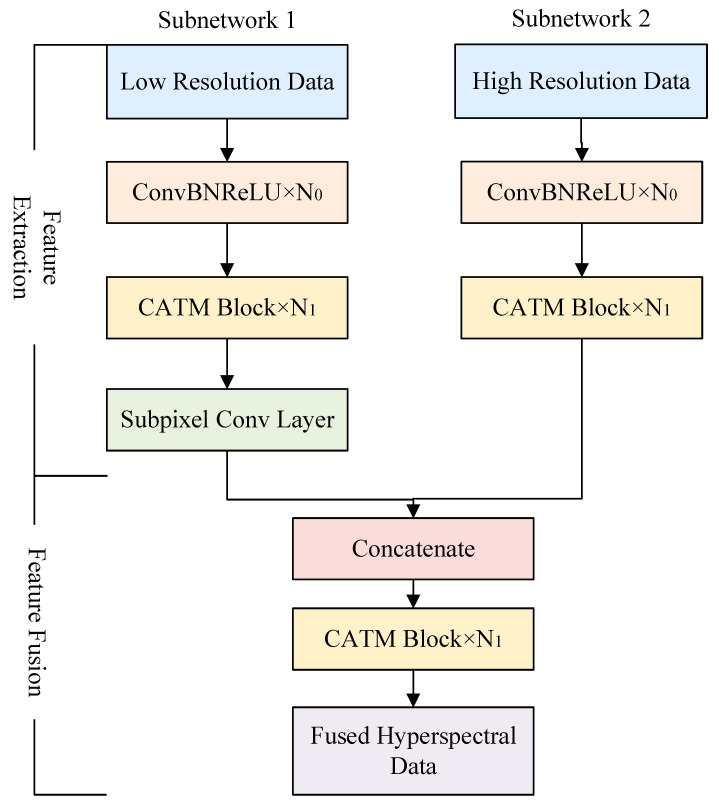
Overall framework.

**Figure 2:**
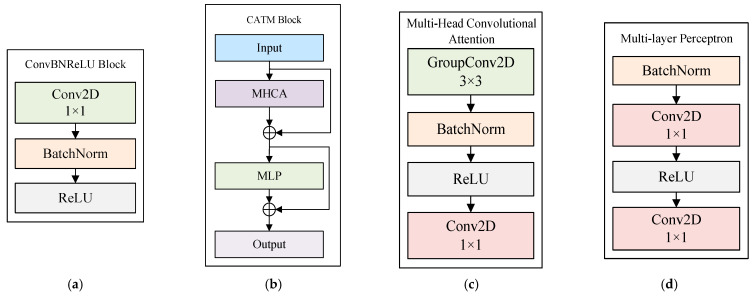
Block architectures. (**a**) ConvBNReLU block, (**b**) CATM block, (**c**) MHCA block, (**d**) MLP block.

The overall framework of the algorithm is shown in Figure 1 below. The overall framework consisted of two branches of sub-networks, the inputs of which were low-resolution data and high-resolution data. Additionally, the overall framework was divided into two parts, the feature extraction part and the feature fusion part. The feature extraction part was divided into the ConvBNReLU block, the convolutional-attention-based token mixer block, and a pixel-shuffle layer for low-resolution data feature extraction sub-branches. The structures of different blocks are shown in Figure 2. The feature fusion part consisted of the concatenate layer and the CATM block to better fuse the extracted information from each data. The number of each block was set to be N_0_ and N_1_.

### 2.1. ConvBNReLU Block

The ConvBNReLu block was placed on the top of the network to extract shallow spatial–spectral information. It consisted of a 1 × 1 convolution layer, a BatchNormalization layer, and a ReLu activation layer.

### 2.2. Convolutional-Attention-Based Token Mixer Block

The BottleNeck block introduced by ResNet [1] has been a prominent feature in visual neural networks due to its inherent inductive biases and compatibility with various hardware platforms. However, it falls short in terms of effectiveness when compared to the transformer block. The transformer block has demonstrated outstanding performance across a range of visual tasks, with its exceptional capabilities attributed to the combination of the MetaFormer paradigm [2] and the attention-based token mixer module [3]. Nevertheless, the transformer block lags behind the BottleNeck block in terms of inference speed, primarily due to its intricate attention mechanisms, posing challenges in many real-world industrial applications.

For remote sensing images, it is important to focus on the local information and context information of an area. Thus, we referred to the idea of the next convolution block in Li et al.’s work [4] to better capture the short-term dependencies. The CATM block followed the structure of MetaFormer, which consisted of the token mixer module as an efficient token mixer with a deployment-friendly convolution operation and the Multi-layer Perceptron (MLP) module within the framework of MetaFormer.

(1) $$x^{l}=MHCA\left(x^{l-1}\right)+x^{l-1}$$

(2) $$x^{l}=MLP\left(x^{l-1}\right)+x^{l-1}$$

### 2.3. Multi-Head Convolutional Attention Block (MHCA)

In order to obtain local representation learning, we used the structure of the multi-head convolutional attention block. Following the structure of multi-head self-attention, the multi-head convolutional attention block also used the multi-head paradigm to build convolutional attention. The local information can be learned from different representation subspaces at different positions. The structure of MHCA is shown in Figure 2. It can be represented as:

(3) $$x^{l}=Concat(GC\left(x^{1}\right),GC\left(x^{2}\right),\ldots ,GC(x^{n}))W_{P}$$

The ${x=[x}^{1},x^{2},\ldots ,x^{n}]$ indicates dividing the input feature x into the n-parallelled multi-head form in the channel dimension. To promote the information interaction across the multiple heads, we also equipped MHCA with a projection layer (WP). GC was single-head convolutional attention, which can be defined as:

(4) $$GC\left(x\right)=O(W,{(T}_{m},T_{n}))$$

in which O represents the inner product operation with trainable parameters W, and ${(T}_{m},T_{n})$ represents adjacent tokens in input feature x. GC is capable of learning the affinities between different tokens in the local receptive field through iteratively optimizing trainable parameter W. Additionally, we used BatchNormalization (BN) and ReLU to achieve normalization.

### 2.4. Subpixel Convolutional Layer

**Figure 3:**
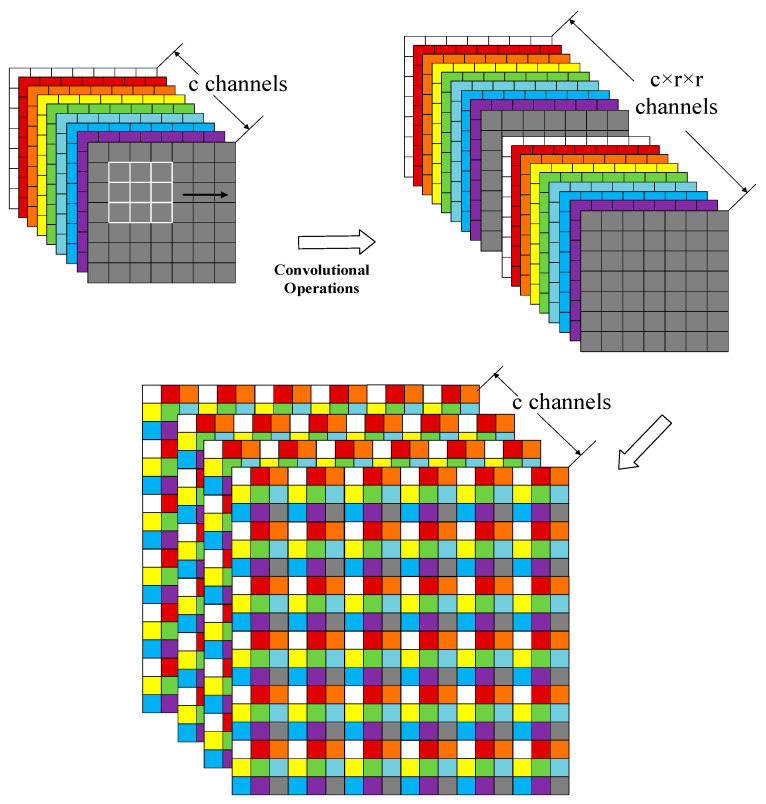
Subpixel convolutional layer.

As the resolutions between different data inputs were disparate, we needed to upsample the coarse data information so as to fuse both pieces of data on the same scale. Thus, we used the pixel shuffle layer originating from the sub-pixel convolution proposed in [5] to upscale the coarse information. The process is illustrated in Figure 3. This was to convert the channel into $r^{2}$ times the number of original bands, in which r represents the upscaling factor. Then, the channel dimension was adjusted to r by a process that can be represented as:

(5) $$X_{up}=PS(W_{L}*f^{L-1}(X_{low})+b_{L})$$

(6) $$PS(X_{x,y,c})=X_{\left[\frac{x}{r}],[\frac{y}{r}],c\cdot r\cdot mod(y,r)+c\cdot mod(x,r\right)}$$

in which $X_{low}$ represents the input data, $W_{L}$ represents the convolutional operation, and $b_{L}$ represents bias. PS represents PixelShuffle. $X_{x,y,c}$ x,y,c represents the coordinates on x, y, and the channels, and r represents the multiples.

## 3. Experiments and Results

### 3.1. Datasets

For hyperspectral data resolution enhancement, we used a combination of ZY1-02D hyperspectral data with a spatial resolution of 30 m and multispectral data with a spatial resolution of 10 m. Table 1 shows the information on ZY1-02D satellite. The images were obtained at 33°44′9.45″–34°6′22.12″ N and 118°18′11.98″–118°46′6.91″ E. The two images were co-registered by ENVI. For the infrared data resolution enhancement, we used a combination of ZY1-02D multispectral data with a spatial resolution of 10 m and thermal infrared data with a spatial resolution of 16 m. The two datasets mainly contained fields and buildings.

### 3.2. Experimental Settings

We set number N_0_ as 1 and number N_1_ as 3, as they are shown to be relatively balanced for running speed and accuracy. The model training followed the Wald protocol [6], down-sampling multispectral and hyperspectral images three times to the resolutions of 30 and 90 m to obtain the input of the training samples and outputting the original hyperspectral images as training samples. We trained the model by segmenting multispectral images into 96 × 96 patches and hyperspectral images into 32 × 32 patches. During prediction, the original multispectral image and hyperspectral image, both maintained at resolutions of 10 and 30 m, were segmented by the aforementioned size and input into the model for prediction. Finally, the fused hyperspectral image with the improved resolution was restored. We used ENVI to process the georeferencing. By obtaining the map and coordinate information of the whole image, each pixel was given the geoinformation.

The fusion of thermal infrared and multispectral images also adopted the above model, and the model training followed the Wald protocol. The multispectral and thermal infrared images were down-sampled 1.5 times to the resolutions of 15 and 20 m to obtain the input of the training samples, and the original thermal infrared images were used as the output of the training samples. We trained the model by segmenting multispectral images into 96 × 96 blocks and thermal infrared images into 32 × 32 blocks. When making predictions, the original multispectral images and thermal infrared images, both maintained at resolutions of 10 and 15 m, were divided into the aforementioned sizes and input into the model for prediction.

For the infrared data resolution enhancement experiment, we compared our method with the state-of-the-art (SOTA) method, including traditional fusion methods of MTFGLP [7] and SFIM [10], as well as deep learning methods of MSDCNN [11], ConSSFCNN [13], and SSRNet [13]. For the hyperspectral resolution enhancement experiment, we compared our method with SOTA methods, including traditional methods of MAP [14], MTFGLP, and SFIM, as well as deep learning methods of MSDCNN, ConSSFCNN, SSRNet, and TFNet [15]. The reason for the difference in contrasting methods between infrared data and hyperspectral resolution enhancement experiments is that some of the methods are not applicable to infrared, data due to the inherent characteristics of the algorithm. For the quantitative experiment, we chose fusion indices of CC (correlation coefficient) [16], RMSE (root mean square error) [17], ERGAS (Erreur Relative Globale Adimensionnelle de Synthese) [18], PSNR (peak signal noise ratio) [19], and SSMI (structure similarity index measure) [20]. We compared the results in both the visual and quantitative aspects. The experiments were conducted on the CPU of Intel Xeon Bronze 3106 and GPU of NVIDIA Tesla T4.

### 3.3. Results

**Figure 4:**
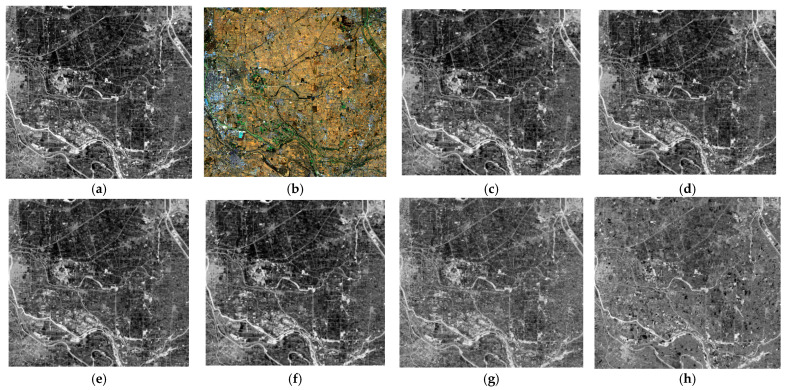
RGB composites of enhancing thermal data via different methods. (**a**) Original infrared data, (**b**) multispectral data, (**c**) SRE-CATM, (**d**) MTFGLP, (**e**) SFIM, (**f**) MSDCNN, (**g**) ConSSFCNN, and (**h**) SSRNet.

**Figure 5:**
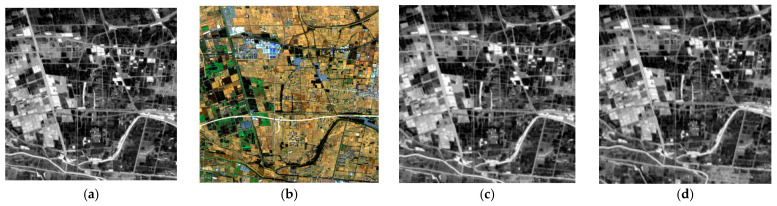

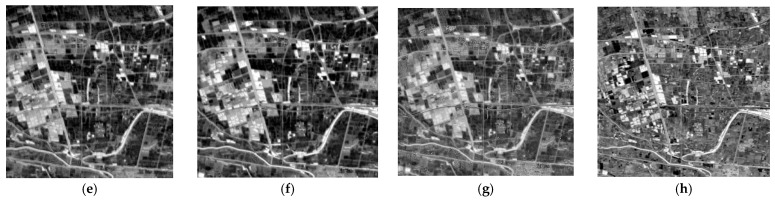
RGB composites of enhancing thermal data via different methods (in detail). (**a**) Original infrared data, (**b**) multispectral data, (**c**) SRE-CATM, (**d**) MTFGLP, (**e**) SFIM, (**f**) MSDCNN, (**g**) ConSSFCNN, and (**h**) SSRNet.

For the infrared data resolution enhancement, we compared our method with five different state-of-the-art methods, which included MTFGLP, SFIM, MSDCNN, ConSSFCNN, and SSRNet. Figure 4 and Figure 5 show the visual comparison in whole and in detail, with ours showing clear texture and resemblance. Table 2 shows the quality indices results of the visual-thermal data fusion. The results showed that our method outperformed other SOTA methods in both spatial and overall domains.

**Figure 6:**
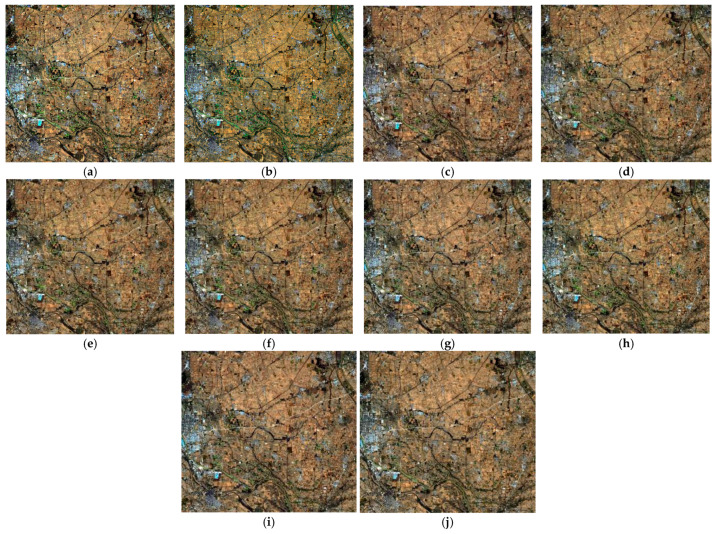
RGB composites of enhancing hyperspectral data via different methods. (**a**) Original hyperspectral, (**b**) multispectral, (**c**) SRE-CATM, (**d**) MAP, (**e**) MTFGLP, (**f**) SFIM, (**g**) MSDCNN, (**h**) ConSSFCNN, (**i**) SSRNet, and (**j**) TFNet.

**Figure 7:**
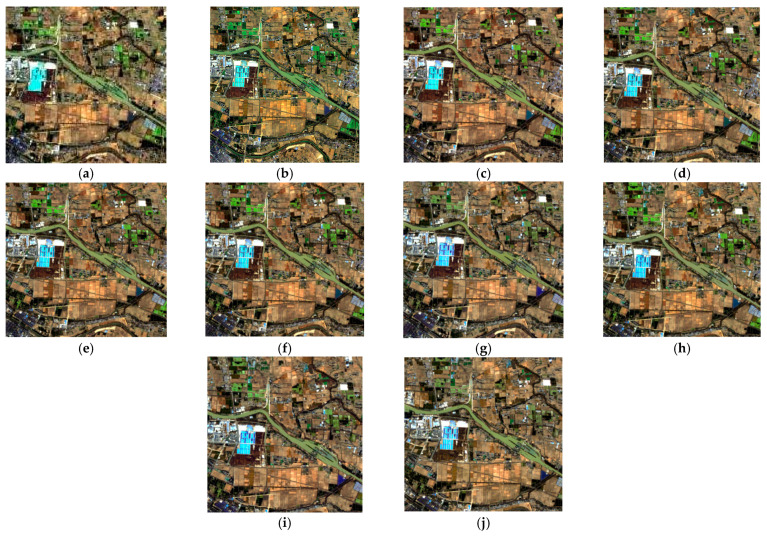
RGB composites of enhancing hyperspectral data via different methods (in detail). (**a**) Original hyperspectral, (**b**) multispectral, (**c**) SRE-CATM, (**d**) MAP, (**e**) MTFGLP, (**f**) SFIM, (**g**) MSDCNN, (**h**) ConSSFCNN, (**i**) SSRNet, and (**j**) TFNet.

For the hyperspectral data resolution enhancement, we compared our method with seven different state-of-the-art methods, which included MAP, MTFGLP, SFIM, MSDCNN, ConSSFCNN, SSRNet, and TFNet. Figure 6 and Figure 7 show the visual comparison, with ours showing clear texture and tone. Table 3 shows the quality indices results of hyperspectral data fusion. The results showed that our method outperformed most other SOTA methods in both spatial and spectral domains, although it was slightly inferior to TFNet in the spatial texture similarity.

**Figure 8:**
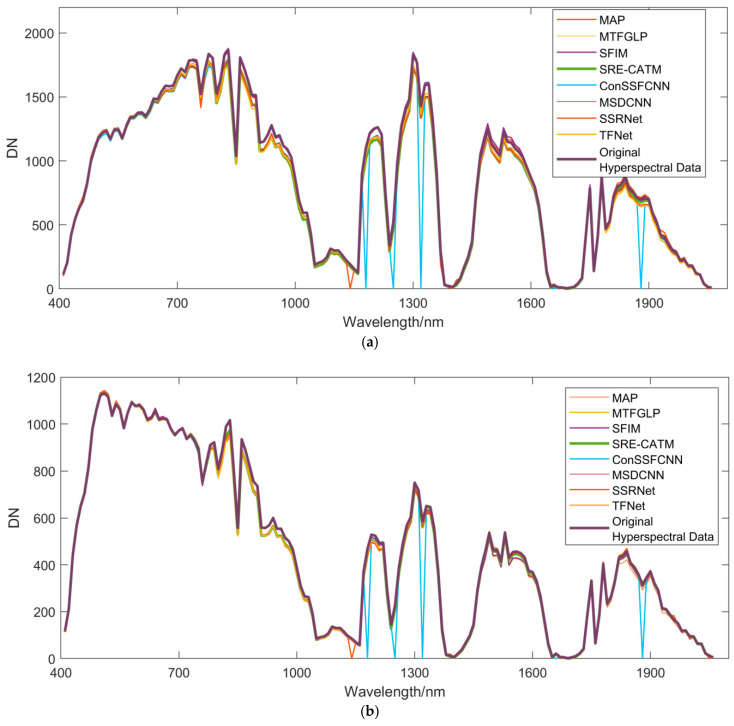

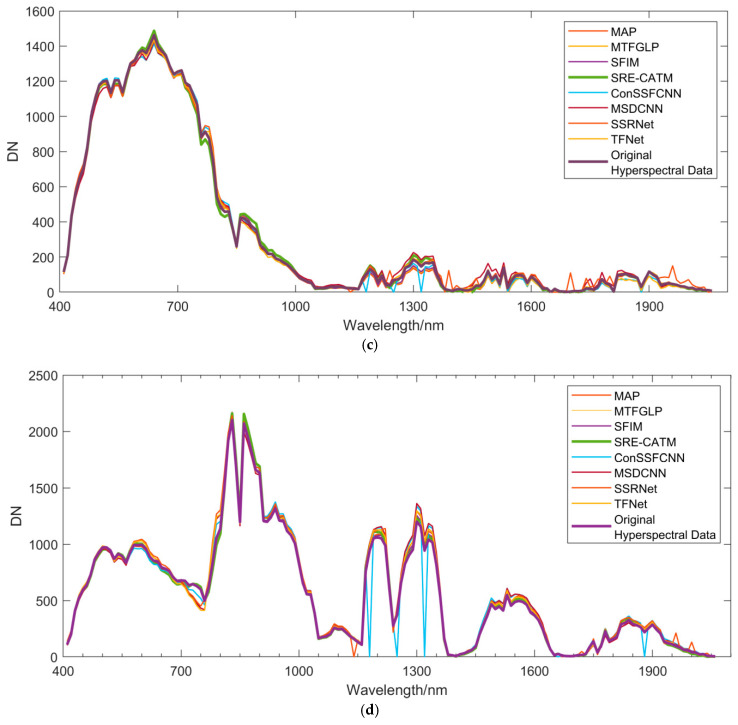
Spectra of different landcover types using different methods. (**a**) Bare soil, (**b**) roads, (**c**) water, and (**d**) fields.

Additionally, we chose four typical landcovers to extract their spectra on different fusion results, as shown in Figure 8. We can see that all methods can restore the basic form of the original hyperspectral data.

SRE-CATM outperformed traditional methods, due to the advantages of deep learning—it was more robust and had more capacity to learn the association between spatial and spectral features. The reason for SRE-CATM outperforming other deep learning methods may due to its effective design of being an attentional token mixer and the subpixel layer, which does not involve upscaling or downscaling operations, which, for some SOTAs, it is needed to conduct outside the algorithm and may cause deviation.

### 3.4. Discussion

In this study, we proposed the SRE-CATM method, which utilized the convolutional attention token mechanism to better improve the spatial resolution of remote sensing images of hyperspectral and infrared data. Consistent with previous studies of enhancing low-resolution data’s spatial resolution by undermining the spatial and spectral relationships between high-spatial resolution data and low-spatial resolution data, our method did not use transformer directly but following the MetaFormer paradigm, replacing the token mixer as the multi-head convolutional attention. This greatly improved the efficiency and operability and was more suitable for industrial deployment. SRE-CATM may greatly improve the accuracies in object detection, land classification, etc., that require a higher spatial resolution that current single data cannot obtain yet.

## 4. Conclusions

In this article, we proposed a spatial resolution enhancement framework using the convolutional visual transformer. The method used the transformer block and sub-pixel convolution to extract spatial and spectral information and fused them using the same technique. The convolutional transformer block can effectively utilize the local information of spatial and spectral dimensions. The method was tested on two kinds of data types, which included the visual-thermal dataset and visual-hyperspectral dataset. Our method was also compared with the state-of-the-art methods, including traditional methods and deep learning methods. The experiment results showed that the method was effective and outperformed state-of-the-art methods in overall, spatial, and spectral accuracies. This method can effectively improve the spatial resolution of hyperspectral or infrared thermal images, which can improve the accuracies of real-world applications by providing fine texture extraction.

## Figures and Tables

**Table 1:** Information on ZY1-02D satellite.

Sensor	Parameter		
Hyperspectral Camera	Spectral Range		0.4~2.5 μm
	Number of Bands		166
	Ground Pixel Resolution		30 m
	Swath		60 km
	Spectral Resolution	Visible Near-infrared	10 nm, 76 bands
		ShortWave infrared	20 nm, 90 bands
Visible/near infrared camera	Spectral Range	Panchromatic	B01: 0.452~0.902 μm
		Multispectral	B02: 0.452~0.521 μm B03: 0.522~0.607 μm B04: 0.635~0.694 μm B05: 0.776~0.895 μm B06: 0.416~0.452 μm B07: 0.591~0.633 μm B08: 0.708~0.752 μm B09: 0.871~1.047 μm
	Ground Pixel Resolution		Panchromatic: 2.5 m Multispectral: 10 m
	Swath		115 km
Thermal infrared camera	Spectral range		8~10 μm
	Ground pixel resolution		≤16 m
	Swath Width		≥115 km
	Nedt (K)		≤0.2 (@300 K Black body)
	Dynamic Range (K)		240~340 (Black body)

**Table 2:** Quantitative results of enhancing thermal data.

	SRE-CATM	MTFGLP	SFIM	MSDCNN	ConSSFCNN	SSRNet
CC	**0.9991**	0.9605	0.9400	0.9754	0.8312	0.5650
RMSE	**4.1877**	8.1071	10.0496	8.5416	18.5567	23.9213
ERGAS	**0.0700**	0.2706	0.3354	0.6433	0.6186	1.7928
PSNR	**56.2174**	50.1392	48.3192	49.3037	43.0788	38.7311
SSMI	**0.9954**	0.7308	0.6518	0.8641	0.5457	0.4589

The bold represents highest results.

**Table 3:** Quantitative results of enhancing hyperspectral data.

	SRE-CATM	MAP	MTFGLP	SFIM	MSDCNN	ConSSFCNN	SSRNet	TFNet
CC	**0.9893**	0.9468	0.9492	0.9319	0.9779	0.9779	0.9244	0.9727
SAM	**0.6970**	1.2541	1.0403	1.0823	0.8917	0.8917	1.3064	0.9446
RMSE	**13.6138**	20.8091	15.3219	242.9297	14.4426	14.4426	18.0121	15.1021
ERGAS	**1.2387**	3.6650	3.7916	17.6060	1.5351	1.5351	5.5100	1.6189
PSNR	**45.9485**	43.5238	44.7663	45.9772	44.4024	44.4024	43.4039	44.1445
SSMI	0.9693	0.8375	0.8857	0.8753	0.9698	0.9698	0.9406	**0.9732**

The bold represents highest results.

## Data Availability

The data are not publicly available due to data restrictions.
